# Ultrapure Dialysis Fluid: A New Standard for Contemporary Hemodialysis

**DOI:** 10.5812/numonthly.3060

**Published:** 2012-06-20

**Authors:** Bernard Canaud, Paungpaga Lertdumrongluk

**Affiliations:** 1 Nephrology, Dialysis and Intensive Care Unit, Montpellier, France

**Keywords:** Renal Dialysis, Renal Replacement Therapy

## 1. Introduction

The concept of renal replacement therapy (RRT) has evolved considerably over the last 2 decades. Dialysis, a key component of RRT intended to clear uremic toxins and periodically restore the internal milieu composition, has benefited from considerable advances in dialysis technology (bicarbonate-buffered dialysis fluid, ultrafiltration-controlled systems, profiling systems, blood volume and temperature control, direct quantification, and high-flux dialyzers) and innovative adjunctive drug therapies designed to correct anemia (erythropoietinstimulating agents (ESA), IV iron, etc.), metabolic bone disease (vitamin D and analogs, calcimimetics, etc.), and associated metabolic disorders (lipid-lowering agents, antioxidants, etc.) (1). Such refinement in optimizing RRT would have not been possible without intense and collaborative clinical research, which led to a better understanding of uremic complications and improvement in the standards of care for chronic kidney disease patients (2).

Despite these medical and technical advances, it is disappointing to note that morbidity and mortality still remain high in dialysis-dependent chronic kidney disease patients (3). Most recent studies have noted that the dialysis population has changed over the last decade, characterized by patients who are older and suffer from multiple comorbid conditions, including diabetes and cardiovascular diseases that compromise patient outcomes (4). Indeed, it has also been shown that chronic microinflammation represents the common link and amplifying factor to such dialysis-related pathology (5, 6). In this interesting debate, it is strange to note that the nephrology community has overlooked the microbial purity of dialysis fluid while technical solutions to correct impure dialysis fluid have been available for 2 decades (7, 8). This study is intent on supporting the use of ultrapure dialysis fluid (UPDF) in all hemodialysis (HD) modalities and showing that UPDF is technically and economically feasible in most dialysis facilities worldwide. (9, 10).

## 2. UPDF as a Surrogate for Sterile and Non-Pyrogenic Dialysis Fluid

The term “ultrapure” was coined in the early 80s to underline the fact that dialysis fluid solutions (water and dialysis fluid) were highly purified in comparison to standard procedures and were used as a surrogate for sterile and non-pyrogenic fluid (11). UPDF was defined as containing < 0.1 colony-forming unit/ml (CFU/ml) using sensitive microbiological methods and < 0.03 endotoxin unit/ml (EU/ml) using the limulus amoebocyte lysate (lAl) assay. This definition is now widely accepted and used for UPDF determination in international guidelines. A summary of microbiological standards for water and dialysis fluids in HD is given in Table 1.

**Table 1:** Microbiological Standards for Water and Dialysis Fluid Purity

	**Standard Water**	**Standard Dialysate**	**Ultrapure Water**	**Ultrapure Dialysate**	**Sterile Dialysate**
Bacterial limits a, CFU/mL	< 100-200	< 100-200	< 0.1	< 0.1	< 10-6
Endotoxin limits b, EU/mL	< 0.25-2	< 0.25	< 0.03	< 0.03	< 0.03

^a^Adequate monitoring and microbiological technique (i.e. UPDF,poor media TGEA, R2A,17-23ºC,7 days)

^b^Sensitive LAL assay, threshold detection limit , 0.03 EU/mL

## 3. UPDF is Easily Produced by Online Cold Sterilization

**Figure 1:**
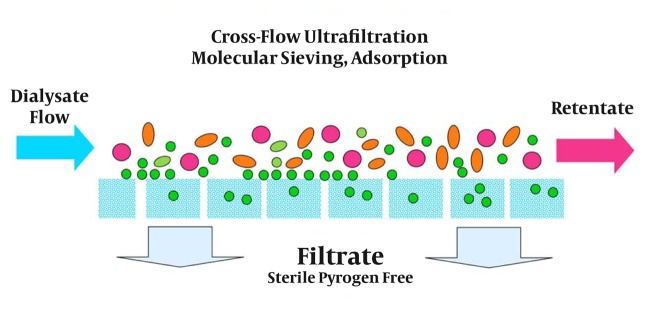
Cold Sterilization Process Based on Ultrafiltration

**Figure 2:**
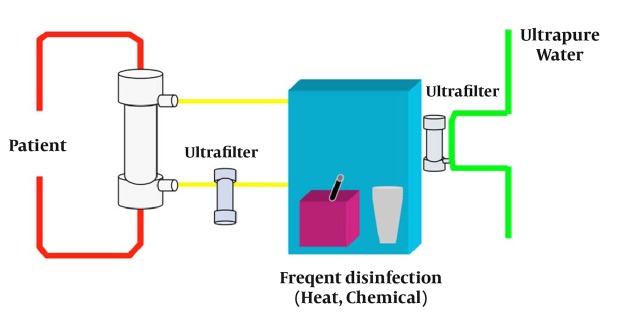
HD Machine Equipped With Sterilizing Ultrafilters

Technical aspects of producing UPDF have been described in detail elsewhere (12). UPDF relies on 3 basic principles: use of ultrapure water; installation of sterilizing ultrafilters (1 or 2) in the dialysis fluid pathway on adequately designed HD machines; and implementation of strict hygienic rules (disinfection procedures and ultrafilter changes) and regular microbiological monitoring (13). Figure 1 presents the concept of the cold sterilization process based on tangential ultrafiltration. Figure 2 shows HD machines equipped with ultrafilters installed in series, designed to ensure a final cold sterilization of the dialysis fluid flowing into the patient’s hemodialyzer.

## 4. UPDF is Justified by Operative Conditions of Contemporary Dialysis

HD has emerged as a leading component of this innovation. hemodialyzer membranes have improved in performance, resulting in a major increase in solute removal capacities for both small and middle molecules (high-flux membranes), and a significant improvement in biocompatibility (synthetic high-flux membranes) (14). hemodialyzer performance has also improved, thanks to new geometry designs favoring back-transport phenomena and convective clearance imposed by ultrafiltration controller systems installed onto dialysis machines (15). Along these lines, the microbiological purity of dialysis fluid has become a critical component, recognized as a key element in the HD biocompatibility network (16). Standards of purity for water and dialysis fluid established in the 70s were later recognized as being poorly adapted to the setting of contemporary HD conditions (17). A recent transcontinental agreement (Europe, US, Japan) has recognized the need to upgrade water and dialysis fluid purity for all dialysis modalities. For this purpose, guidelines supporting the regular use of UPDF for all HD modalities and editing handling and hygienic rules have been established (18-20). Beneficial effects of regular use of UPDF are seen in intermediate and long-term outcomes in dialysis patients (21).

## 5. UPDF Prevents Inflammation and Its Deleterious Biological and Clinical Consequences

Intermediate outcomes are mainly related to the preventive and/or anti-inflammatory effects associated with the regular use of UPDF (22). Several controlled and/or randomized studies have demonstrated that UPDF use was accompanied by a decrease in sensitive inflammatory markers (21) and sustained reduction of chronic inflammation (23) in HD patients. Interestingly, correction of the microinflammatory state is associated with better correction of anemia and decreased requirements for ESA)(24-26), suggesting better ESA responsiveness (27, 28).

In addition, the use of UPDF is associated with a reduction in plasma levels of beta-2 microglobulin and pentosidine (29). Myeloperoxidase activity and lipid profile tend to improve in parallel with CRP reduction in patients exposed to UPDF (30-32). Monocyte activation and apoptosis and the release of inflammatory factors are reduced with the use of UPDF (33). In addition, oxidative stress is minimized with the combination of high-flux membrane and UPDF (34). Residual renal function is better preserved over a 24-month period in the UPDF-treated group, as shown in a randomized controlled trial (35, 36). Nutritional status and visceral protein levels improved significantly in a UPDF-treated group, compared to their counterparts treated with conventional (contaminated) dialysis fluid (37, 38).

## 6. UPDF Reduces Morbidity and Mortality in HD Patients

Beneficial effects of UPDF on morbidity and mortality of dialysis patients are more difficult to ascertain because there are several confounding factors (39). The use of synthetic high-flux membranes and enhanced convective clearance by online hemodiafiltration (ol-HDF) facilitating the removal of middle- and large-molecular-weight uremic toxins are the two most prominent factors (40, 41). Indeed, using UPDF with more efficient modalities (ol-HDF or high-flux HD) should not be considered exclusion criteria but rather an incentive, and there is strong support for its generalization in dialysis (21).

The use of UPDF is associated with significant reduction in morbidity (42) and cardiovascular events (43). In addition, in a recent randomized controlled trial, locatelli et al. have shown that by combining the use of UPDF and convective therapies (HF and HDF), the incidence of hypotensive episodes could be significantly reduced (44).

Interestingly, 2 retrospective cohort studies have reported a dramatic reduction in the prevalence of beta-2 microglobulin amyloidosis, as revealed by carpal tunnel syndrome surgery, with extended use of UPDF and synthetic membranes (26, 45). One study also reported significant improvement in the painful and disabling symptomatology of beta-2 microglobulin amyloidosis after switching conventional dialysate with UPDF (46). In a retrospective cohort study, cardiovascular morbidity and mortality was decreased (47) in dialysis patients mainly exposed to UPDF.

ol-HDF, which represents the most advanced dialysis modality and requires the use of UPDF, is associated with better outcomes for dialysis patients. In most recent cohort studies, the use of high-efficiency (high-volume) HDF was associated with a relative risk reduction of all-cause mortality averaging 35% (48, 49). Interestingly, cardiovascular mortality was particularly reduced in 2 recent studies underlining the potential beneficial effects of UPDF and convective therapies on the vascular disease of dialysis patients (50, 51). All these studies underlined the fact that ultrapurity of the dialysis fluid was the common factor of improvement, mediated through a reduction of the chronic microinflammatory status of dialysis patients.

## 7. UPDF is Technically and Economically Feasible for All Dialysis Facilities

Several reports have shown that UPDF was accessible and affordable in most dialysis centers (52). In this perspective, the recent intermediary analysis of the CoN-TRAST study is the most significant (53). Ten dialysis facilities were selected for conducting the water and dialysis fluid microbiological audit. Precise and sensitive microbial monitoring of water and dialysis fluid, including bacteriometry (nutrient-poor media (R2A) culture over 7 days) and endotoxin content (limulus amoebocyte lysate using a chromogenic method), were performed monthly over the 2-year period of follow-up. of the 3961 dialysis fluid samples, 99.1% complied with the ultrapurity standard as defined by European Best Practice Guidelines and Dutch guidelines. No side effects or pyrogenic effects were noted in 97 patients who received 11258 ol-HDF sessions. In brief, this study confirms that UPDF may be easily produced on a country-wide scale and used in virtually all contemporary dialysis facilities.

Economic issues associated with the regular use of UPDF should not be ignored and kept under a veil of silence. The production of ultrapure water requires a water treatment system (WTS), including pretreatment (softener, activated carbon, microfiltration), a water polishing system (based on a double reverse-osmosis system in series), and a well-designed distribution loop (ensuring permanent circulation of water with immediate delivery to dialysis machines). Disinfection processes (type, agent, and frequency) and microbiological monitoring of WTS are established according to contamination levels and facility practices. HD machines should be equipped with captive ultrafilters, ensuring a final cold sterilization of the dialysis fluid produced. HD machines are disinfected after each run and ultrafilters are replaced according to manufacturer recommendations. Microbiological monitoring of dialysis fluid is performed periodically according to local and regulatory practices. Considering the fact that ultrapure water is a standard for newly created dialysis facilities in Europe, the only additional cost is associated with the periodic changes of sterilizing ultrafilters installed on the HD machines and the microbiological monitoring of dialysis fluids. Based on a rigorous cost analysis conducted over the last 5 years in our units, we estimated this extra cost at 5 euros per session. The additional costs generated by this high standard of water and dialysis fluid purity are offset by direct and indirect clinical benefits, including better correction of anemia with reduced ESA, and improved patient outcomes with reduced morbidity and hospitalization rates (46). It would be interesting to conduct an economic prospective study on HD patients treated with UPDF to precisely evaluate cost savings in terms of ESA dose, nutritional improvement, and hospitalization reduction.

## 8. UPDF Will Be the Basic Requirement for Developing Innovative Dialysis Modalities for Future Renal Replacement Therapy

In the perspective of developing or improving future dialysis methods, such as ol-HDF (enhanced internal HDF, hemofiltration, etc.), automated dialysis machines ensuring dialyzer priming and rinsing (home and/or self-care machines), and biofeedback-controlled machines (pulse IV infusion, volume control, etc.), it seems obvious that UPDF will be a basic resource for such development. Considering the high-quality refinement of dialysis fluids, we must deduce that UPDF offers a more efficient barrier against proinflammatory biological reactions, at no risk to dialysis patients.

To conclude, UPDF must be considered a basic component of contemporary HD therapy for preventing chronic inflammation and improving patient outcomes in high-flux HD. The use of UPDF is an additional step required to develop ol-HDF and related innovative renal replacement therapies (47).
